# The genome sequence of the Dogs-Mercury Flea Beetle, *Hermaeophaga mercurialis *(Fabricius, 1792)

**DOI:** 10.12688/wellcomeopenres.22896.1

**Published:** 2024-09-03

**Authors:** Liam M. Crowley, Mark Telfer, Maxwell V. L. Barclay, Dominic Phillips, University of Oxford and Wytham Woods Genome Acquisition Lab, Natural History Museum Genome Acquisition Lab, Darwin Tree of Life Barcoding collective, Wellcome Sanger Institute Tree of Life Management, Samples and Laboratory team, Wellcome Sanger Institute Scientific Operations: Sequencing Operations, Wellcome Sanger Institute Tree of Life Core Informatics team, Tree of Life Core Informatics collective, Darwin Tree of Life Consortium

**Affiliations:** 1 University of Oxford, Oxford, England, UK; 2 Entomological Consultant, Ventnor, Isle of Wight, England, UK; 3 Natural History Museum, London, England, UK

**Keywords:** Hermaeophaga mercurialis, Dogs-Mercury Flea Beetle, genome sequence, chromosomal, Coleoptera

## Abstract

We present a genome assembly from an individual Dogs-Mercury Flea Beetle, *Hermaeophaga mercurialis* (Arthropoda; Insecta; Coleoptera; Chrysomelidae). The genome sequence has a length of 479.40 megabases. Most of the assembly is scaffolded into 9 chromosomal pseudomolecules. The mitochondrial genome has also been assembled and is 16.05 kilobases in length. Gene annotation of this assembly on Ensembl identified 12,633 protein-coding genes.

## Species taxonomy

Eukaryota; Opisthokonta; Metazoa; Eumetazoa; Bilateria; Protostomia; Ecdysozoa; Panarthropoda; Arthropoda; Mandibulata; Pancrustacea; Hexapoda; Insecta; Dicondylia; Pterygota; Neoptera; Endopterygota; Coleoptera; Polyphaga; Cucujiformia; Chrysomeloidea; Chrysomelidae; Galerucinae; Alticini; Hermaeophagina; *Hermaeophaga*; *Hermaeophaga mercurialis* (Fabricius, 1792) (NCBI:txid347362).

## Background

*Hermaeophaga mercurialis* (Fabricius, 1792), or Dog’s-Mercury Flea Beetle (Coleoptera: Chrysomelidae: Galerucinae: Alticini: *Hermaeophaga*), is a small species of flea beetle measuring between 2.3 and 4.0 mm ( Duff, 2016). *H. mercurialis* possesses a broad, dorsally convex body with a head largely concealed beneath the pronotal fore margin. It is a flightless species with vestigial wings and has a spur on each tibia ( Duff, 2016; UK Beetles, 2024). *H. mercurialis* has a blue-black body with black appendages; however, the colour of the antennomeres varies. The basal half of antennomere 1 is dark brown, while the apical half along with antennomeres 2 to 4 are orange-brown, and the tarsi are rust coloured ( Duff, 2016; UK Beetles, 2024).

*Hermaeophaga mercurialis* adults occur year-round, overwintering among leaf litter, under bark, and within grass tussocks. They become active early in the year, reaching a peak in abundance from May to June and can remain active until late autumn ( Duff, 2016; UK Beetles, 2024). Adults mate early in the season, and eggs are laid within the base soil of host plants. The larvae emerge after approximately 10 weeks and bore into the roots of the host plant or feed on basal leaves. Once mature, larvae burrow into the soil to pupate ( Duff, 2016; UK Beetles, 2024). *H. mercurialis* feeds on the leaves of a single host plant in the UK, *Mercurialis perennis* (Dog’s Mercury); however, it has also been recorded on closely related species in central and southern Europe ( Duff, 2016; UK Beetles, 2024).

*H. mercurialis* is widespread in southern England, while it is local in south Wales, and central and north-west England. There are some older records in northern Wales and sparse records for Scotland and Northern Ireland ( GBIF Secretariat, 2024; NBN Atlas Partnership, 2024; UK Beetles, 2024). Globally, it is considered a widespread Palearctic species, mostly distributed in central and southern Europe, spreading across to Asia Minor, through Ukraine and Western Russia, and upwards to southern Sweden ( GBIF Secretariat, 2024; NBN Atlas Partnership, 2024; UK Beetles, 2024). *H. mercurialis* can be found in most habitats where the host plant ( *Mercurialis perennis*) is abundant, namely woodland, grassland, heathland, and hedgerows.

*Mercurialis perennis*, the predominant host plant for *H. mercurialis*, is an important indicator species for ancient woodland health and can be used to determine how long a woodland has been established ( Peterken & Game, 1981; Schmidt *et al.*, 2014; Slaviero *et al.*, 2011). Ancient woodland is one of the rarest habitat types in the UK. *M. perennis* is quick to colonise the floors of ancient woodland. *H. mercurialis* assists in the natural control of *M. perennis*, consuming the poisonous toxins produced by the plant. The continued release of species barcodes through the UK Barcode of Life project ( Price *et al.*, 2023), along with the full genome sequencing of *H. mercurialis* by the DToL project, will contribute to a better understanding of the relationships between indicator species and their associated insects.

## Genome sequence report

**Figure 1.**
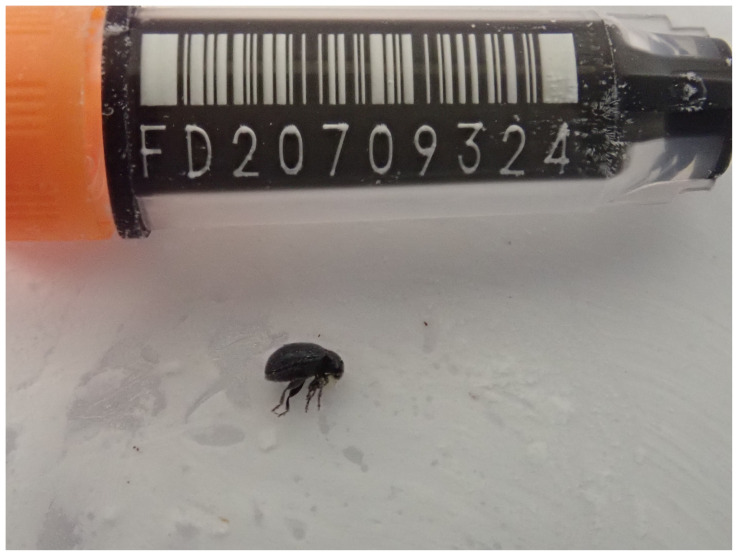
Photograph of the *Hermaeophaga mercurialis* (icHerMerc2) specimen used for genome sequencing.

The genome of an adult *Hermaeophaga mercurialis* ( Figure 1) was sequenced using Pacific Biosciences single-molecule HiFi long reads, generating a total of 20.08 Gb (gigabases) from 2.32 million reads, providing approximately 39-fold coverage. Primary assembly contigs were scaffolded with chromosome conformation Hi-C data, which produced 116.40 Gbp from 770.84 million reads, yielding an approximate coverage of 243-fold. Specimen and sequencing information is summarised in Table 1.

**Table 1.** Specimen and sequencing data for *Hermaeophaga mercurialis*.

Project information			
**Study title**	*Hermaeophaga mercurialis* (dogs-mercury flea beetle)		
**Umbrella BioProject**	PRJEB57105		
**Species**	*Hermaeophaga mercurialis*		
**BioSample**	SAMEA8603245		
**NCBI taxonomy ID**	347362		
Specimen information			
**Technology**	**ToLID**	**BioSample accession**	**Organism part**
**PacBio long read sequencing**	icHerMerc2	SAMEA8603880	Whole organism
**Hi-C sequencing**	icHerMerc3	SAMEA8603881	Whole organism
**RNA sequencing**	icHerMerc5	SAMEA111458369	Thorax and abdomen
Sequencing information			
**Platform**	**Run accession**	**Read count**	**Base count (Gb)**
**Hi-C Illumina NovaSeq 6000**	ERR10446382	7.71e+08	116.4
**PacBio Sequel IIe**	ERR10439747	2.32e+06	20.08
**RNA Illumina NovaSeq 6000**	ERR12245528	5.85e+07	8.84

**Figure 2.**
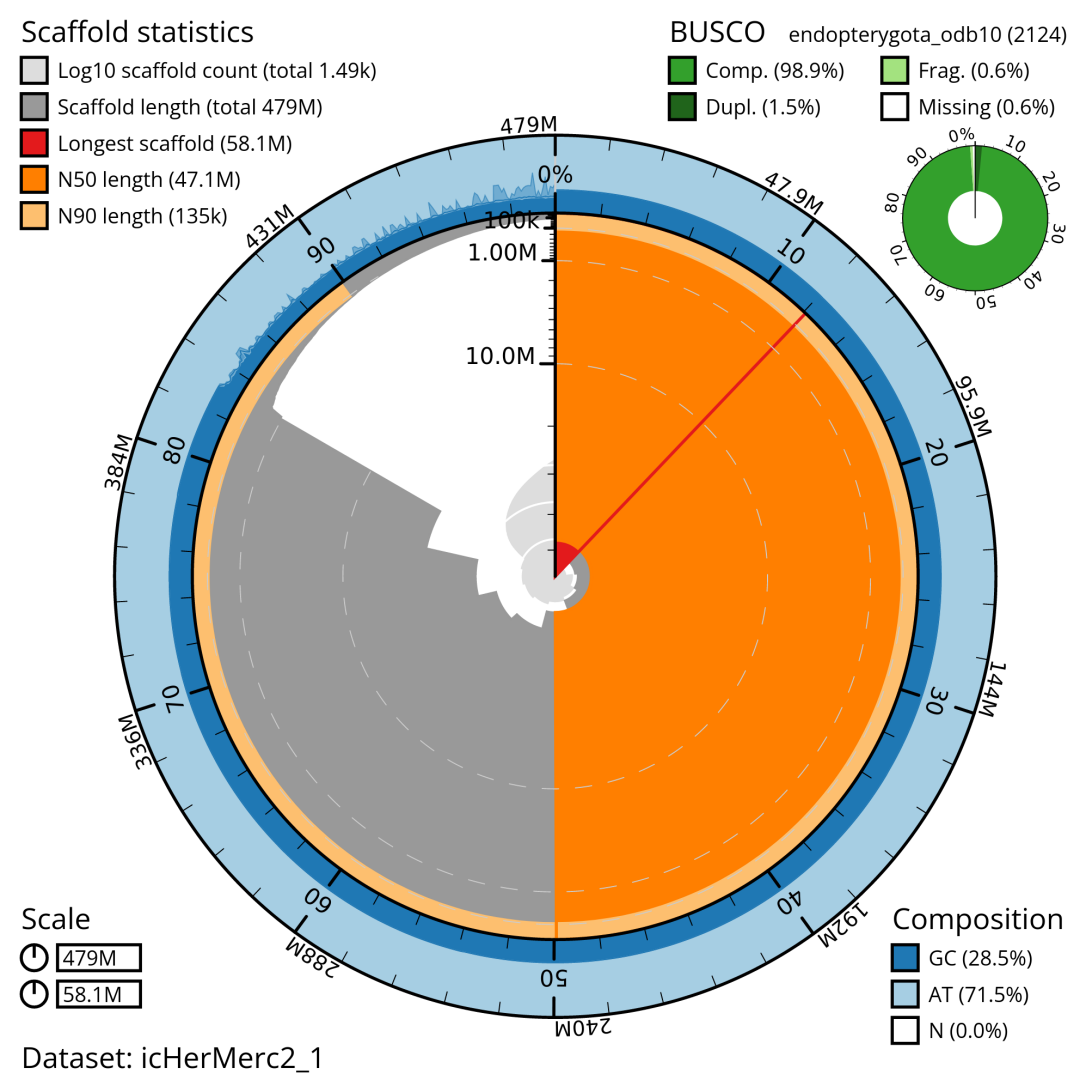
Genome assembly of *Hermaeophaga mercurialis*, icHerMerc2.1: metrics. The BlobToolKit snail plot shows N50 metrics and BUSCO gene completeness. The main plot is divided into 1,000 size-ordered bins around the circumference with each bin representing 0.1% of the 479,410,748 bp assembly. The distribution of scaffold lengths is shown in dark grey with the plot radius scaled to the longest scaffold present in the assembly (58,096,663 bp, shown in red). Orange and pale-orange arcs show the N50 and N90 scaffold lengths (47,101,683 and 135,186 bp), respectively. The pale grey spiral shows the cumulative scaffold count on a log scale with white scale lines showing successive orders of magnitude. The blue and pale-blue area around the outside of the plot shows the distribution of GC, AT and N percentages in the same bins as the inner plot. A summary of complete, fragmented, duplicated and missing BUSCO genes in the endopterygota_odb10 set is shown in the top right. An interactive version of this figure is available at https://blobtoolkit.genomehubs.org/view/icHerMerc2_1/dataset/icHerMerc2_1/snail.

**Figure 3.**
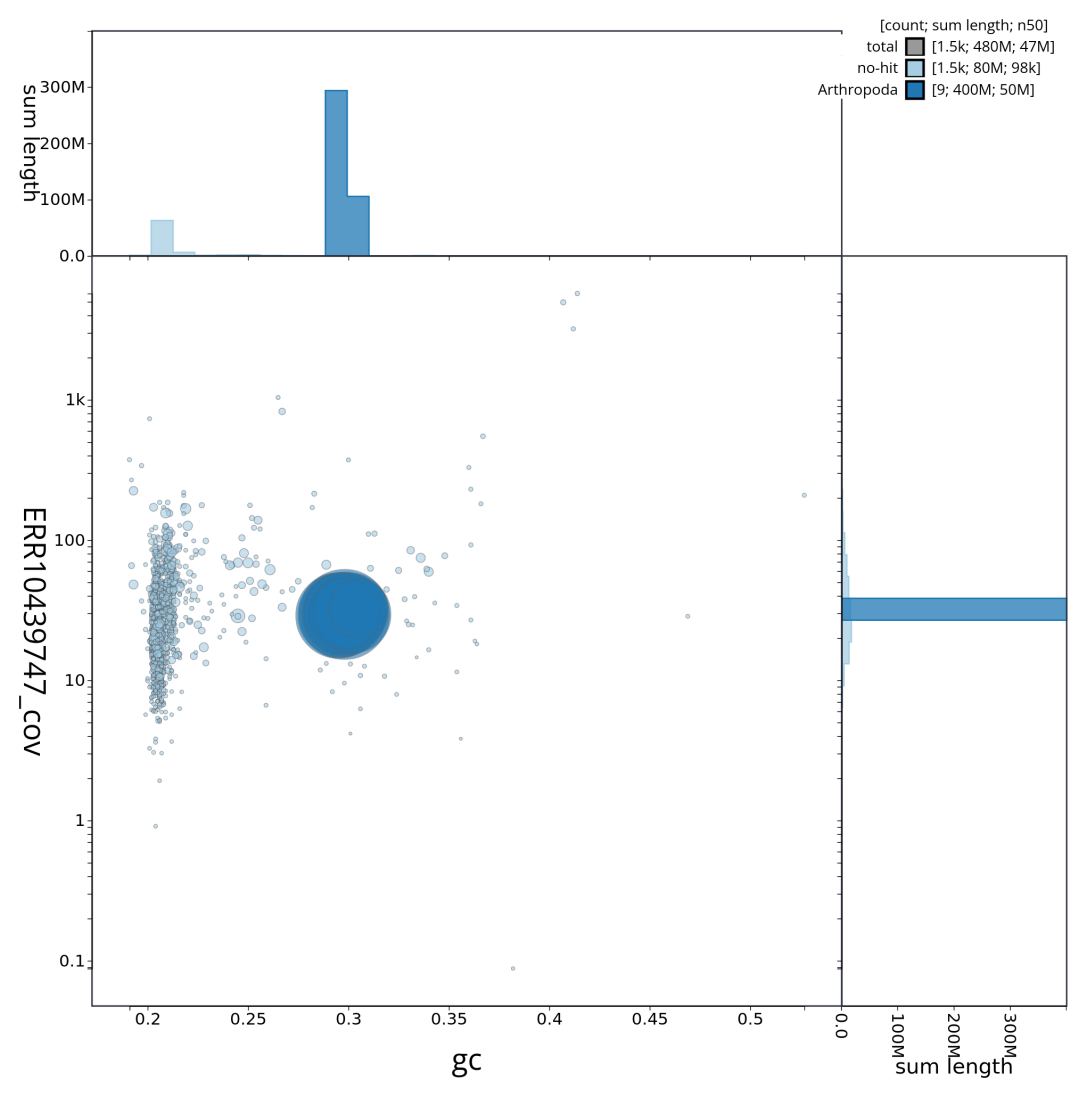
Genome assembly of *Hermaeophaga mercurialis*, icHerMerc2.1: Blob plot of base coverage in ERR10439747 against GC proportion for sequences in assembly icHerMerc2.1. Sequences are coloured by phylum. Circles are sized in proportion to sequence length. Histograms show the distribution of sequence length sum along each axis. An interactive version of this figure is available at https://blobtoolkit.genomehubs.org/view/icHerMerc2_1/dataset/icHerMerc2_1/blob.

**Figure 4.**
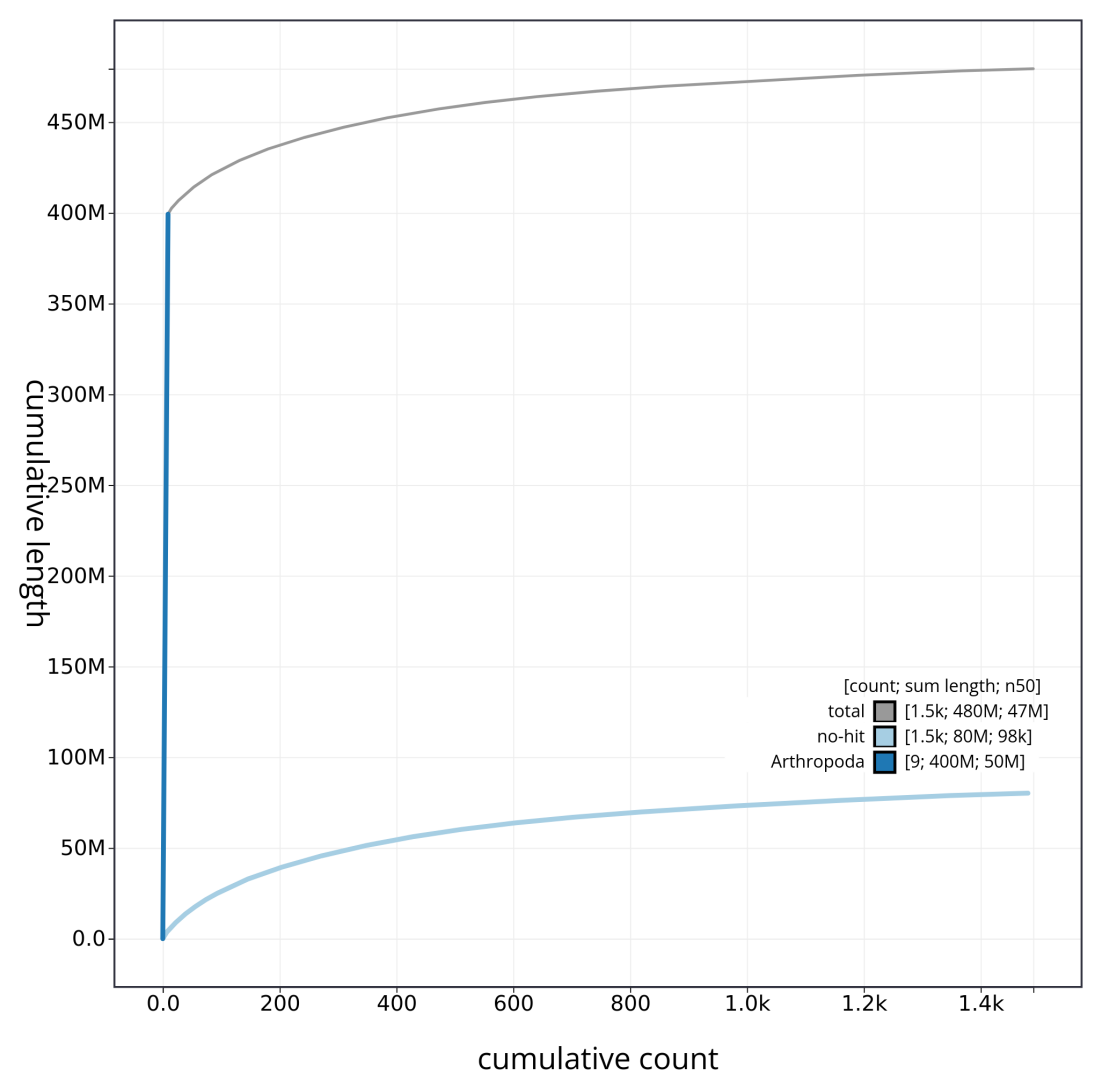
Genome assembly of *Hermaeophaga mercurialis* icHerMerc2.1: BlobToolKit cumulative sequence plot. The grey line shows cumulative length for all sequences. Coloured lines show cumulative lengths of sequences assigned to each phylum using the buscogenes taxrule. An interactive version of this figure is available at https://blobtoolkit.genomehubs.org/view/icHerMerc2_1/dataset/icHerMerc2_1/cumulative.

**Figure 5.**
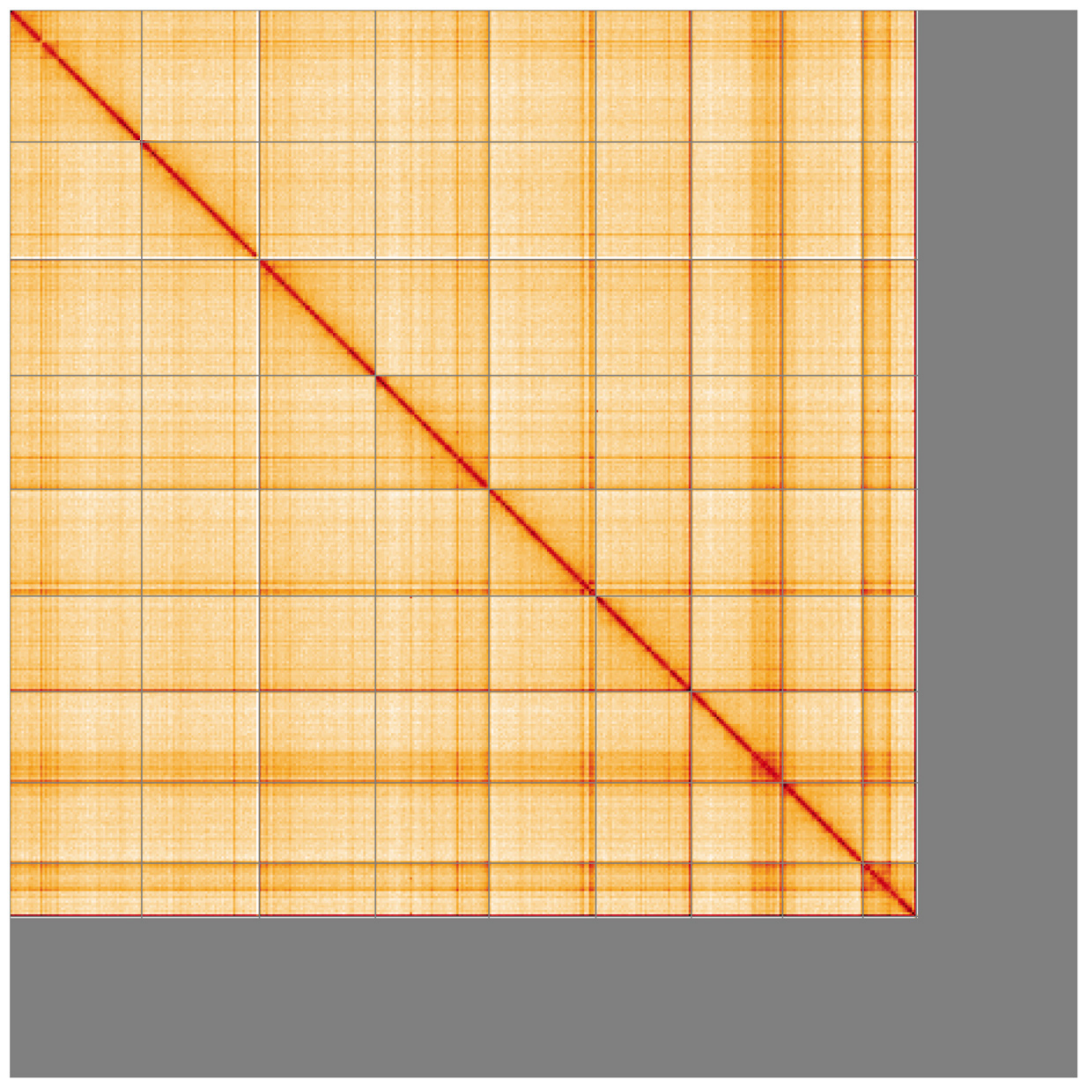
Genome assembly of *Hermaeophaga mercurialis* icHerMerc2.1: Hi-C contact map of the icHerMerc2.1 assembly, visualised using HiGlass. Chromosomes are shown in order of size from left to right and top to bottom. An interactive version of this figure may be viewed at https://genome-note-higlass.tol.sanger.ac.uk/l/?d=M5t2W6dSSUupdH6Lpr1Phw.

Manual assembly curation corrected 15 missing joins or mis-joins, reducing the scaffold number by 0.67%, and increasing the scaffold N50 by 8.02%. The final assembly has a total length of 479.40 Mb in 1,489 sequence scaffolds, with 416 gaps. The scaffold N50 is 47.1 Mb ( Table 2). The snail plot in Figure 2 provides a summary of the assembly statistics, while the distribution of assembly scaffolds on GC proportion and coverage is shown in Figure 3. The cumulative assembly plot in Figure 4 shows curves for subsets of scaffolds assigned to different phyla. Most (83.48%) of the assembly sequence was assigned to 9 chromosomal-level scaffolds. Chromosome-scale scaffolds confirmed by the Hi-C data are named in order of size ( Figure 5; Table 3). The sex chromosomes could not be determined. While not fully phased, the assembly deposited is of one haplotype. Contigs corresponding to the second haplotype have also been deposited. The mitochondrial genome was also assembled and can be found as a contig within the multifasta file of the genome submission.

**Table 2.** Genome assembly data for *Hermaeophaga mercurialis*, icHerMerc2.1.

Genome assembly		
Assembly name	icHerMerc2.1	
Assembly accession	GCA_951812935.1	
*Accession of alternate haplotype*	*GCA_951812905.1*	
Span (Mb)	479.40	
Number of contigs	1,906	
Contig N50 length (Mb)	1.4	
Number of scaffolds	1,489	
Scaffold N50 length (Mb)	47.1	
Longest scaffold (Mb)	58.1	
Assembly metrics *		*Benchmark*
Consensus quality (QV)	64.2	*≥ 50*
*k*-mer completeness	100.0%	*≥ 95%*
BUSCO **	C:98.9%[S:97.4%,D:1.5%],F:0.6%,M:0.6%,n:2,124	*C ≥ 95%*
Percentage of assembly mapped to chromosomes	83.48%	*≥ 95%*
Sex chromosomes	Not identified	*localised homologous pairs*
Organelles	Mitochondrial genome: 16.05 kb	*complete single alleles*
Genome annotation of assembly GCA_951812935.1 at Ensembl		
Number of protein-coding genes	12,633	
Number of non-coding genes	1,206	
Number of gene transcripts	21,120	

* Assembly metric benchmarks are adapted from column VGP-2020 of “Table 1: Proposed standards and metrics for defining genome assembly quality” from Rhie *et al.* (2021). ** BUSCO scores based on the endopterygota_odb10 BUSCO set using version 5.3.2. C = complete [S = single copy, D = duplicated], F = fragmented, M = missing, n = number of orthologues in comparison. A full set of BUSCO scores is available at https://blobtoolkit.genomehubs.org/view/icHerMerc2_1/dataset/icHerMerc2_1/busco.

**Table 3.** Chromosomal pseudomolecules in the genome assembly of *Hermaeophaga mercurialis*, icHerMerc2.

INSDC accession	Name	Length (Mb)	GC%
OX638358.1	1	58.1	30.0
OX638359.1	2	51.83	29.5
OX638360.1	3	51.07	29.5
OX638361.1	4	50.02	30.0
OX638362.1	5	47.1	30.0
OX638363.1	6	42.11	30.0
OX638364.1	7	40.14	30.0
OX638365.1	8	35.4	30.0
OX638366.1	9	23.51	30.5
OX638367.1	MT	0.02	21.0

The estimated Quality Value (QV) of the final assembly is 64.2 with *k*-mer completeness of 100.0%, and the assembly has a BUSCO v5.3.2 completeness of 98.9% (single = 97.4%, duplicated = 1.5%), using the endopterygota_odb10 reference set ( *n* = 2,124).

Metadata for specimens, BOLD barcode results, spectra estimates, sequencing runs, contaminants and pre-curation assembly statistics are given at https://links.tol.sanger.ac.uk/species/347362.

## Genome annotation report

The *Hermaeophaga mercurialis* genome assembly (GCA_951812935.1) was annotated at the European Bioinformatics Institute (EBI) on Ensembl Rapid Release. The resulting annotation includes 21,120 transcribed mRNAs from 12,633 protein-coding and 1,206 non-coding genes ( Table 2; https://rapid.ensembl.org/Hermaeophaga_mercurialis_GCA_951812935.1/Info/Index). The average transcript length is 12,458.48. There are 1.53 coding transcripts per gene and 6.10 exons per transcript.

## Methods

### Sample acquisition and DNA barcoding

The specimens of *Hermaeophaga mercurialis* used for PacBio HiFi sequencing (specimen ID Ox001027, ToLID icHerMerc2) and Hi-C sequencing (specimen ID Ox001028, ToLID icHerMerc3) were collected from Wytham Woods, Oxfordshire (biological vice-county Berkshire), UK (latitude 51.77, longitude –1.31) on 2020-12-08 by tussocking. The specimens were collected by Liam Crowley (University of Oxford) and identified by Mark Telfer (independent researcher) and preserved on dry ice.

The specimen used for RNA sequencing (specimen ID NHMUK014433935, ToLID icHerMerc5) was collected from Naphill Common, England, UK (latitude 51.67, longitude –0.79) on 2021-05-02 by hand picking. The specimen was collected and identified by Maxwell Barclay (Natural History Museum) and preserved by dry freezing at –80 °C.

The initial species identification was verified by an additional DNA barcoding process according to the framework developed by Twyford *et al*. (2024). A small sample was dissected from each specimen and stored in ethanol, while the remaining parts were shipped on dry ice to the Wellcome Sanger Institute (WSI). The tissue was lysed, the COI marker region was amplified by PCR, and amplicons were sequenced and compared to the BOLD database, confirming the species identification ( Crowley *et al.*, 2023). Following whole genome sequence generation, the relevant DNA barcode region was also used alongside the initial barcoding data for sample tracking at the WSI ( Twyford *et al.*, 2024). The standard operating procedures for Darwin Tree of Life barcoding have been deposited on protocols.io ( Beasley *et al.*, 2023).

### Nucleic acid extraction

The workflow for high molecular weight (HMW) DNA extraction at the WSI Tree of Life Core Laboratory includes a sequence of core procedures: sample preparation and homogenisation, DNA extraction, fragmentation, and clean-up. Protocols developed by the WSI Tree of Life laboratory are publicly available on protocols.io ( Denton *et al.*, 2023b).

In sample preparation, the icHerMerc2 sample was weighed and dissected on dry ice ( Jay *et al.*, 2023). Tissue from the whole organism was homogenised using a PowerMasher II tissue disruptor ( Denton *et al.*, 2023a). HMW DNA was extracted using the Automated MagAttract v1 protocol ( Sheerin *et al.*, 2023). DNA was sheared into an average fragment size of 12–20 kb in a Megaruptor 3 system ( Todorovic *et al.*, 2023). Sheared DNA was purified by solid-phase reversible immobilisation, using AMPure PB beads to eliminate shorter fragments and concentrate the DNA ( Strickland *et al.*, 2023). The concentration of the sheared and purified DNA was assessed using a Nanodrop spectrophotometer and Qubit Fluorometer using the Qubit dsDNA High Sensitivity Assay kit. Fragment size distribution was evaluated by running the sample on the FemtoPulse system.

RNA was extracted from thorax and abdomen tissue of icHerMerc5 in the Tree of Life Laboratory at the WSI using the RNA Extraction: Automated MagMax™ *mir*Vana protocol ( do Amaral *et al.*, 2023). The RNA concentration was assessed using a Nanodrop spectrophotometer and a Qubit Fluorometer using the Qubit RNA Broad-Range Assay kit. Analysis of the integrity of the RNA was done using the Agilent RNA 6000 Pico Kit and Eukaryotic Total RNA assay.

### Sequencing

Pacific Biosciences HiFi circular consensus DNA sequencing libraries were constructed according to the manufacturers’ instructions. Poly(A) RNA-Seq libraries were constructed using the NEB Ultra II RNA Library Prep kit. DNA and RNA sequencing was performed by the Scientific Operations core at the WSI on Pacific Biosciences Sequel IIe (HiFi) and Illumina NovaSeq 6000 (RNA-Seq) instruments. Hi-C data were also generated from whole organism tissue of icHerMerc3 using the Arima-HiC v2 kit. The Hi-C sequencing was performed using paired-end sequencing with a read length of 150 bp on the Illumina NovaSeq 6000 instrument.

### Genome assembly, curation and evaluation

***Assembly***

The HiFi reads were first assembled using Hifiasm ( Cheng *et al.*, 2021) with the --primary option. Haplotypic duplications were identified and removed using purge_dups ( Guan *et al.*, 2020). The Hi-C reads were mapped to the primary contigs using bwa-mem2 ( Vasimuddin *et al.*, 2019). The contigs were further scaffolded using the provided Hi-C data ( Rao *et al.*, 2014) in YaHS ( Zhou *et al.*, 2023) using the --break option. The scaffolded assemblies were evaluated using Gfastats ( Formenti *et al.*, 2022), BUSCO ( Manni *et al.*, 2021) and MERQURY.FK ( Rhie *et al.*, 2020).

The mitochondrial genome was assembled using MitoHiFi ( Uliano-Silva *et al.*, 2023), which runs MitoFinder ( Allio *et al.*, 2020) and uses these annotations to select the final mitochondrial contig and to ensure the general quality of the sequence.

***Assembly curation***

The assembly was decontaminated using the Assembly Screen for Cobionts and Contaminants (ASCC) pipeline (article in preparation). Manual curation was primarily conducted using PretextView ( Harry, 2022), with additional insights provided by JBrowse2 ( Diesh *et al.*, 2023) and HiGlass ( Kerpedjiev *et al.*, 2018). Scaffolds were visually inspected and corrected as described by Howe *et al*. (2021). Any identified contamination, missed joins, and mis-joins were corrected, and duplicate sequences were tagged and removed. The entire process is documented at https://gitlab.com/wtsi-grit/rapid-curation (article in preparation).

***Evaluation of the final assembly***

A Hi-C map for the final assembly was produced using bwa-mem2 ( Vasimuddin *et al.*, 2019) in the Cooler file format ( Abdennur & Mirny, 2020). To assess the assembly metrics, the *k*-mer completeness and QV consensus quality values were calculated in Merqury ( Rhie *et al.*, 2020). This work was done using the “sanger-tol/readmapping” ( Surana *et al.*, 2023a) and “sanger-tol/genomenote” ( Surana *et al.*, 2023b) pipelines. The genome readmapping pipelines were developed using the nf-core tooling ( Ewels *et al.*, 2020), use MultiQC ( Ewels *et al.*, 2016), and make extensive use of the Conda package manager, the Bioconda initiative ( Grüning *et al.*, 2018), the Biocontainers infrastructure ( da Veiga Leprevost *et al.*, 2017), and the Docker ( Merkel, 2014) and Singularity ( Kurtzer *et al.*, 2017) containerisation solutions. The genome was also analysed within the BlobToolKit environment ( Challis *et al.*, 2020) and BUSCO scores ( Manni *et al.*, 2021; Simão *et al.,* 2015) were calculated.

Table 4 contains a list of relevant software tool versions and sources.

**Table 4.** Software tools: versions and sources.

Software tool	Version	Source
BlobToolKit	4.2.1	https://github.com/blobtoolkit/blobtoolkit
BUSCO	5.3.2	https://gitlab.com/ezlab/busco
Hifiasm	0.16.1-r375	https://github.com/chhylp123/hifiasm
HiGlass	1.11.6	https://github.com/higlass/higlass
Merqury	MerquryFK	https://github.com/thegenemyers/MERQURY.FK
MitoHiFi	2	https://github.com/marcelauliano/MitoHiFi
PretextView	0.2	https://github.com/wtsi-hpag/PretextView
purge_dups	1.2.3	https://github.com/dfguan/purge_dups
sanger-tol/genomenote	v1.0	https://github.com/sanger-tol/genomenote
sanger-tol/readmapping	1.1.0	https://github.com/sanger-tol/readmapping/tree/1.1.0
YaHS	yahs-1.1.91eebc2	https://github.com/c-zhou/yahs

### Genome annotation

The Ensembl Genebuild annotation system ( Aken *et al.*, 2016) was used to generate annotation for the *Hermaeophaga mercurialis* assembly (GCA_951812935.1) in Ensembl Rapid Release at the EBI. Annotation was created primarily through alignment of transcriptomic data to the genome, with gap filling via protein-to-genome alignments of a select set of proteins from UniProt ( UniProt Consortium, 2019).

### Wellcome Sanger Institute – Legal and Governance

The materials that have contributed to this genome note have been supplied by a Darwin Tree of Life Partner. The submission of materials by a Darwin Tree of Life Partner is subject to the **‘Darwin Tree of Life Project Sampling Code of Practice’**, which can be found in full on the Darwin Tree of Life website here. By agreeing with and signing up to the Sampling Code of Practice, the Darwin Tree of Life Partner agrees they will meet the legal and ethical requirements and standards set out within this document in respect of all samples acquired for, and supplied to, the Darwin Tree of Life Project. 

Further, the Wellcome Sanger Institute employs a process whereby due diligence is carried out proportionate to the nature of the materials themselves, and the circumstances under which they have been/are to be collected and provided for use. The purpose of this is to address and mitigate any potential legal and/or ethical implications of receipt and use of the materials as part of the research project, and to ensure that in doing so we align with best practice wherever possible. The overarching areas of consideration are:

•   Ethical review of provenance and sourcing of the material

•   Legality of collection, transfer and use (national and international) 

Each transfer of samples is further undertaken according to a Research Collaboration Agreement or Material Transfer Agreement entered into by the Darwin Tree of Life Partner, Genome Research Limited (operating as the Wellcome Sanger Institute), and in some circumstances other Darwin Tree of Life collaborators.

## Data Availability

European Nucleotide Archive: *Hermaeophaga mercurialis* (dogs-mercury flea beetle). Accession number PRJEB57105; https://identifiers.org/ena.embl/PRJEB57105 ( Wellcome Sanger Institute, 2023). The genome sequence is released openly for reuse. The *Hermaeophaga mercurialis* genome sequencing initiative is part of the Darwin Tree of Life (DToL) project. All raw sequence data and the assembly have been deposited in INSDC databases. Raw data and assembly accession identifiers are reported in Table 1 and Table 2.

## Author information

Members of the University of Oxford and Wytham Woods Genome Acquisition Lab are listed here: https://doi.org/10.5281/zenodo.12157525.

Members of the Natural History Museum Genome Acquisition Lab are listed here: https://doi.org/10.5281/zenodo.12159242.

Members of the Darwin Tree of Life Barcoding collective are listed here: https://doi.org/10.5281/zenodo.12158331

Members of the Wellcome Sanger Institute Tree of Life Management, Samples and Laboratory team are listed here: https://doi.org/10.5281/zenodo.12162482.

Members of Wellcome Sanger Institute Scientific Operations: Sequencing Operations are listed here: https://doi.org/10.5281/zenodo.12165051.

Members of the Wellcome Sanger Institute Tree of Life Core Informatics team are listed here: https://doi.org/10.5281/zenodo.12160324.

Members of the Tree of Life Core Informatics collective are listed here: https://doi.org/10.5281/zenodo.12205391.

Members of the Darwin Tree of Life Consortium are listed here: https://doi.org/10.5281/zenodo.4783558.
