# Supplementary material for: The genome sequence of the Dogs-Mercury Flea Beetle, Hermaeophaga mercurialis (Fabricius, 1792)
Source: Wellcome Open Res. 2024 Sep 3;9:503. [Version 1] doi: 10.12688/wellcomeopenres.22896.1 (PMC11489834; doi:10.12688/wellcomeopenres.22896.1)
Supplement: Peer review history

# Reviewer report: Reviewer response for version 1

**DOI:** 10.21956/wellcomeopenres.25212.r103145

**Published:** 2024-10-18

**Authors:** Jerome H L Hui

**Affiliations:** 1 The Chinese University of Hong Kong, Hong Kong, Hong Kong

In this data note, Crowley and colleagues obtained the genomic resource of a flea beetle *Hermaeophaga mercurialis* (Fabricius, 1792) **(Coleoptera; Chrysomelidae). Molecular data of this species are scarce prior to this report, and are mainly mitochondrial and ribosomal gene markers deposited to the NCBI database. Therefore, this new and high-quality genome resource will be useful for further studies, ranging from revealing their population (genetic) structure, dissecting host plant-insect relationships, to understanding their evolution with other insects.

 This genome resource is excellent from the summary statistics, with high BUSCO number scores, high sequence continuity (scaffold N50), and majority of sequences contained on 9 pseudochromosomes (plus mitochondrion). All in all, this is a valuable contribution.

Are sufficient details of methods and materials provided to allow replication by others?

Yes

Is the rationale for creating the dataset(s) clearly described?

Yes

Are the datasets clearly presented in a useable and accessible format?

Yes

Are the protocols appropriate and is the work technically sound?

Yes

Reviewer Expertise:

Genomics, evolution, invertebrates

I confirm that I have read this submission and believe that I have an appropriate level of expertise to confirm that it is of an acceptable scientific standard.

# Reviewer report: Reviewer response for version 1

**DOI:** 10.21956/wellcomeopenres.25212.r101198

**Published:** 2024-10-15

**Authors:** Kali Swichtenberg

**Affiliations:** 1 Arizona State University, Tempe, Arizona, USA

The authors provide a full genome sequenced from Hermaeophaga mercurialis. 

 The specimen photograph is quite low resolution and lacking scale bars. Can a higher resolution photograph using stacked images be provided of a mounted specimen?

 Figure 2 is quite dense and readers may benefit from a written summary of the statistics, especially since the figure demonstrates so many different analyses.

Are sufficient details of methods and materials provided to allow replication by others?

Yes

Is the rationale for creating the dataset(s) clearly described?

Yes

Are the datasets clearly presented in a useable and accessible format?

Yes

Are the protocols appropriate and is the work technically sound?

Yes

Reviewer Expertise:

Single-cell molecular research, symbiont-host cospeciation

I confirm that I have read this submission and believe that I have an appropriate level of expertise to confirm that it is of an acceptable scientific standard.
